# Consensus on Shared Measures of Mobility and Cognition: From the Canadian Consortium on Neurodegeneration in Aging (CCNA)

**DOI:** 10.1093/gerona/gly148

**Published:** 2018-06-21

**Authors:** Manuel Montero-Odasso, Quincy J Almeida, Louis Bherer, Amer M Burhan, Richard Camicioli, Julien Doyon, Sarah Fraser, Susan Muir-Hunter, Karen Z H Li, Teresa Liu-Ambrose, William McIlroy, Laura Middleton, José A Morais, Ryota Sakurai, Mark Speechley, Akshya Vasudev, Olivier Beauchet, Jeffrey M Hausdorff, Caterina Rosano, Stephanie Studenski, Joe Verghese, Canadian Gait and Cognition Network

**Affiliations:** 1 Department of Medicine, Division of Geriatric Medicine, University of Western Ontario, London, Canada; 2 Department of Epidemiology and Biostatistics, Schulich School of Medicine & Dentistry, University of Western Ontario, London, Canada; 3 Gait and Brain Lab, Parkwood Institute, Lawson Health Research Institute, London, Ontario, Canada; 4 Department of Kinesiology and Physical Education, Sun Life Financial Movement Disorders Research Centre, Wilfrid Laurier University, Waterloo, Ontario, Canada; 5 Department of Psychology and PERFORM Centre, Concordia University, Montréal, Quebec, Canada; 6 Centre de Recherche, Institut Universitaire de Gériatrie de Montréal, Quebec, Canada; 7 Department of Medicine, University of Montreal, Quebec, Canada; 8 Montreal Heart Institute, Research Centre, Quebec, Canada; 9 Department of Psychiatry, Geriatric Psychiatry, Schulich School of Medicine, University of Western Ontario, London, Canada; 10 Lawson Health Research Institute, London, Ontario, Canada; 11 Department of Medicine, Geriatric and Cognitive Neurology, University of Alberta, Edmonton, Canada; 12 Functional Neuroimaging Unit, University of Montreal, Quebec, Canada; 13 Interdisciplinary School of Health Sciences, Faculty of Health Sciences, University of Ottawa, Ontario, Canada; 14 Faculty of Health Sciences, School of Physical Therapy, University of Western Ontario, London, Canada; 15 Department of Physical Therapy, Centre for Hip Health and Mobility, University of British Columbia, Canada; 16 Djavad Mowafaghian Centre for Brain Health, Vancouver Coastal Research Institute, University of British Columbia, Canada; 17 Division of Neurology and Department of Medicine, University of Toronto, Ontario, Canada; 18 Tanz Centre for Research in Neurodegenerative Diseases, University of Toronto, Ontario, Canada; 19 Department of Kinesiology, University of Waterloo, Ontario, Canada; 20 Department of Medicine, Division of Geriatrics and Centre of Excellence in Aging and Chronic Disease, McGill University, Montréal, Quebec, Canada; 21 Department of Medicine, Division of Clinical Pharmacology, University of Western Ontario, London, Canada; 22 Department of Medicine, Division of Geriatric Medicine, McGill University, Montréal, Quebec, Canada; 23 Lady Davis Institute for Medical Research, Jewish General Hospital, Montréal, Quebec, Canada; 24 RUIS McGill Centre of Excellence on Aging and Chronic Disease – CEViMaC, Montréal, Quebec, Canada; 25 Center for the Study of Movement, Cognition and Mobility, Neurological Institute, Tel Aviv Sourasky Medical Center, Israel; 26 Department of Physical Therapy, Sackler Faculty of Medicine, and Sagol School of Neuroscience, Tel Aviv University, Israel; 27 Rush Alzheimer’s Disease Center and Department of Orthopedic Surgery, Rush University Medical Center, Chicago, Illinois; 28 Department of Epidemiology, University of Pittsburgh, Pennsylvania; 29 Division of Geriatric Medicine, School of Medicine, University of Pittsburgh, Pennsylvania; 30 Department of Medicine, Albert Einstein College of Medicine, Bronx, New York

**Keywords:** Consensus, Mobility, Cognition, Aging, Gait, Falls, Neurodegenerative diseases

## Abstract

**Background:**

A new paradigm is emerging in which mobility and cognitive impairments, previously studied, diagnosed, and managed separately in older adults, are in fact regulated by shared brain resources. Deterioration in these shared brain mechanisms by normal aging and neurodegeneration increases the risk of developing dementia, falls, and fractures. This new paradigm requires an integrated approach to measuring both domains. We aim to identify a complementary battery of existing tests of mobility and cognition in community-dwelling older adults that enable assessment of motor-cognitive interactions.

**Methods:**

Experts on mobility and cognition in aging participated in a semistructured consensus based on the Delphi process. After performing a scoping review to select candidate tests, multiple rounds of consultations provided structured feedback on tests that captured shared characteristics of mobility and cognition. These tests needed to be sensitive to changes in both mobility and cognition, applicable across research studies and clinics, sensitive to interventions, feasible to perform in older adults, been previously validated, and have minimal ceiling/floor effects.

**Results:**

From 17 tests appraised, 10 tests fulfilled prespecified criteria and were selected as part of the “Core-battery” of tests. The expert panel also recommended a “Minimum-battery” of tests that included gait speed, dual-task gait speed, the Montreal Cognitive Assessment and Trail Making Test A&B.

**Conclusions:**

A standardized assessment battery that captures shared characteristics of mobility and cognition seen in aging and neurodegeneration may increase comparability across research studies, detection of subtle or common reversible factors, and accelerate research progress in dementia, falls, and aging-related disabilities.

Mobility and cognitive impairments often coexist in older adults and are an early phenomenon on the pathway to deficiencies in activities of daily living (1,2). Furthermore, cognitive impairment is more likely to progress to dementia if accompanied by mobility impairment, like slow gait speed (3–6); and similarly, mobility impairment is more likely to result in falls and fractures if accompanied by cognitive impairment (1,7).

Until recently, clinicians and researchers evaluated and managed mobility and cognitive impairments in older adults separately. Approaching these domains as distinct entities obscured the understanding of common underlying mechanisms and the potential for novel integrated treatments (8). For example, cognition could be a target of intervention strategies for mobility improvement or falls prevention, and vice-versa (1,9,10).

The “Motoric Cognitive Risk (MCR) Syndrome” (4,11) and the “Gait and Cognition Syndrome” (3,6,12) are conceptual constructs that combine slow gait with subjective, and slow gait with objective cognitive impairments, respectively, and are associated with an increased risk of developing mobility decline, falls, and dementia (6,13–16). These “syndromes” are commonly observed in older adults and may represent preclinical stages of dementia disorders, less resilience in aging, and prefrailty (2,17–19). Importantly, the coexistence of mobility and cognitive impairments yields the highest risk for mobility decline, including falls and fractures, for dementia syndromes, and even for important adverse outcomes such as mortality. Mechanistically, this coexistence has been associated with central nervous system pathology, even in the absence of overt neurological diseases (1,2). Brain areas and networks specifically involved in gait control and navigation, including the prefrontal cortex and hippocampus, are essential for higher-level cognitive function. These brain areas are susceptible to white matter hyper-intensities burden and cerebral infarcts, and neurodegenerative pathology, which are common findings in older adults prior to developing vascular or neurodegenerative dementias (20).

The conceptual framework for this consensus is represented in Figure 1, which highlights the necessity for integrated measures for risk estimation in clinical and research settings, at preclinical stages of mobility and cognitive decline (1,21). Previous systematic reviews show that not all mobility domains are equally associated with cognition; similarly, not all cognitive domains are equally associated with mobility (18,19). Likewise, some cognitive tests require a higher motor response than others, and some mobility tests require higher-order information processing at the cognitive level. Those tests that capture both cognitive and motor function are said to measure the cognitive-motor interaction, and may be more responsive to subtle decline or better at predicting adverse outcomes than tests that measure only cognitive or mobility domains. Our goal was to propose a battery of tests that captured shared characteristics of mobility and cognition in older adults, feasible to apply, sensitive to interventions, and that could yield comparable results to other studies. Such tests could move the field forward for researchers and clinicians by optimizing the evaluation of the cognitive-motor interaction in older adults.

**Figure 1.**
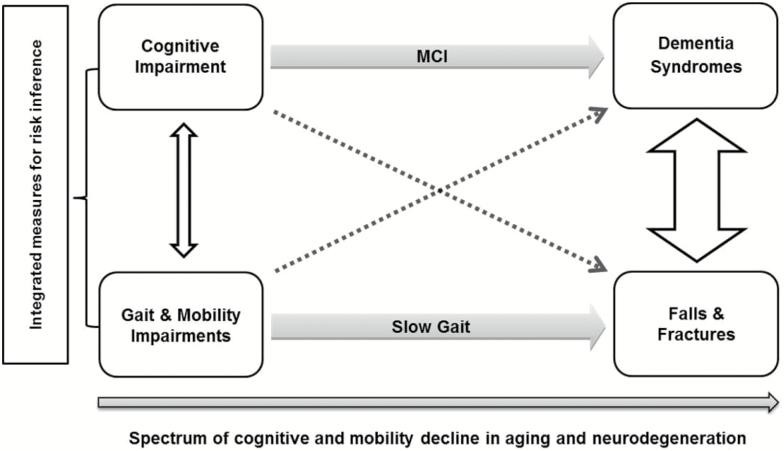
Concurrent decline of cognition and mobility in aging and neurodegeneration. Gray Arrows: cognitive impairment predicts dementia with Mild Cognitive Impairment (MCI) as an intermediate state. Gait impairments increase fall risk and slow gait mediates the association. White Arrows show that cognitive and gait impairments, as well as dementia and falls, are interrelated (arrow thickness represents the strength of associations). Dashed Arrows represent that gait abnormalities (slow gait, high dual-task cost) can predict dementia; similarly, executive and memory dysfunction can predict fall risk. *Note*: DTC = Dual-task gait cost. Adapted from Montero-Odasso et al. (1) and Amboni et al. (6).

Our objectives for this Consensus were the following:

1. To identify mobility and cognitive tests that enable the assessment of motor-cognitive interactions in older adults.
2. To select a “core-battery” of mobility and cognitive tests that could be used as:
  a. Diagnostic and/or prognostic tool for mobility and cognitive decline and incident of dementia syndromes.
  b. Outcome measure for interventional studies aiming to maintain or improve motor-cognitive function.
3. To select a “minimum-battery” from the core set that is feasible to perform in 15 minutes and that could be used in clinical practice and for large-scale or epidemiological studies.

## Methodological Approach

In 2013, 16 Canadian experts were invited to be part of a *Canadian Gait and Cognition Network* by the consensus’ chair (MMO) during the creation of a team for the Canadian Consortium in Neurodegeneration in Aging (CCNA) from March 2013 to November 2013. Experts were selected based on their background as researchers and clinician-scientists affiliated to a Canadian university or Research Institute who had a national and international recognized expertise in the fields of mobility, gait and balance, and cognition. Experts had to have a sustained track record of investigations in the motor-cognitive relationship seen in aging and neurodegeneration. Additionally, five internationally recognized scientists in the field were invited as an ad-hoc advisory board to take part in the rounds of consultations (list of expert members and advisory board are shown in Supplementary Table A).

For this consensus, a semistructured consensus building methodology was used, based on the Delphi process (22). The first step consisted of a scoping literature search done by the consensus’ chair in January 2015, using PubMed database, to select an initial pool of potentially relevant tests, originally designed to measure either mobility or cognition, but that had been shown to be associated which bidirectional changes in both domains and showed associations with progression to dementia, mobility decline, and/or falls. Search strategy included the following terms: mobility[All Fields] AND (“cognition”[MeSH Terms] OR “cognition”[All Fields]) AND (“weights and measures”[MeSH Terms] OR (“weights”[All Fields] AND “measures”[All Fields]) OR “weights and measures”[All Fields] OR “measures”[All Fields]) AND (“gait”[MeSH Terms] OR “gait”[All Fields]) AND (“aged”[MeSH Terms] OR “aged”[All Fields]). After removing duplicates, 102 unique titles remained, 70 were selected by title, and 65 abstracts of relevant articles were screened. Thirty-four articles in full texts were then studied by the chair of the consensus to identify potentially relevant tests. Backwards citation search was also performed.

The second step took place in February 2015, and consisted of circulating a white paper drafted among the experts, by the chair of the consensus. For this asynchronous discussion, experts were asked to complete three tasks within a 30-day window: (a) to perform a literature review and add candidate tests based on whether they showed associations with both mobility and cognition outcomes, including progression to dementia, mobility decline, and/or falls, (b) to identify prespecified criteria to select a final list of core-tests, and (c) to volunteer to present a related topic during the consensus meeting (list of topics and speakers can be found in Supplementary Table A).

The consensus’ chair produced a revised version of the white paper after each round of asynchronous discussion, including comments and any additional tests suggested for appraisal by the experts, during the 30-day window discussion. For the final round, 17 potentially relevant tests had been included in the white paper. The rationale to select these 17 tests over others was based on evidence that suggests that not all cognitive domains are equally associated with mobility, and vice-versa. For instance, global cognition, memory, executive function, and processing speed were found to be more strongly associated with mobility compared with, for instance, visual spatial abilities or verbal fluency (19). And thus, tests evaluating these cognitive domains with proven associations with mobility outcomes were selected. Similarly, gait speed seems to have stronger associations with cognition when compared with grip strength and balance tests, which explains their exclusion (17–19). The list of prespecified criteria identified by the group to aid in the selection of tests is listed in Table 1.

**Table 1.** Prespecified Criteria to Select Measures and Assessment Tools

Criteria	Description
1	Sensitive to changes in both, mobility and cognitive performance
2	No ceiling or floor effects
3	Previously validated in research studies
4	Applicable in both, research and clinics
5	Sensitive to interventions including exercises and cognitive remediation
6	Feasibility: Inexpensive, easy to perform, and minimal expertise required

On March 2015, the final version of the white paper was distributed among the members of the international advisory board, who provided feedback concerning the 17 tests selected and contributed further comments. The final step involved a face-to-face meeting in April 2015 during the 35th Scientific Meeting of the Canadian Geriatrics Society, where 8 expert members of the consensus and 3 members of the advisory board presented background information on motor-cognitive interactions in aging and neurodegeneration, and appraised the 17 candidate tests selected.

**Figure 2.**
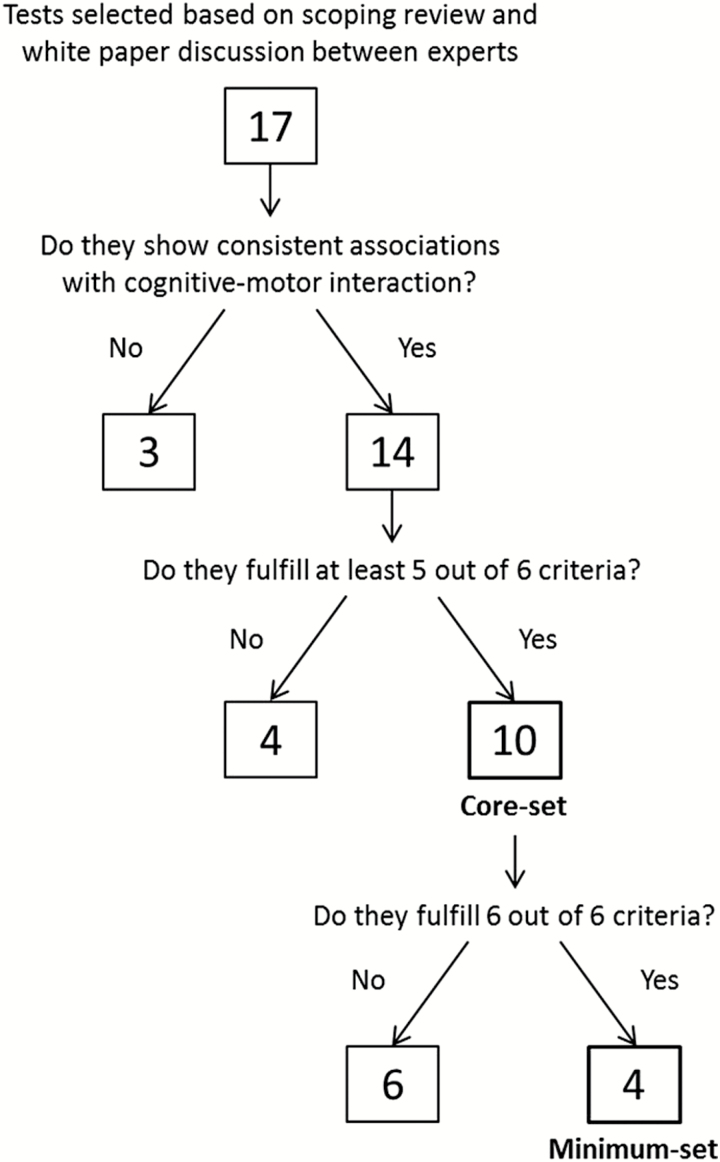
Decision tree showing the flow of the selection of tests by the consensus.

The consensus meeting culminated with a round table discussion where experts and advisory board members applied the set of prespecified criteria previously discussed to narrow down the selection to a core-battery of tests and a minimum-battery of tests to assess both motor and cognitive function (as shown in Figure 2).

## Results

### Mobility Tests Appraised

Mobility tests that underlie areas such as gait performance, balance, and lower limb function were considered (Table 2) based on previous systematic reviews showing consistent associations with cognitive outcomes (4). Measures of additional mobility/motor domains, such as grip strength, were not included in our candidate measures because consistent associations with cognitive outcomes were less robust.

**Table 2.** Mobility Tests Appraised to Evaluate Motor-cognitive Interaction in Aging

Measure	Description	Prespecified Criteria Fulfilled	Advantages	Key Limitations	Clinical Significance of Change	Result
**1. Gait speed**	Individuals walk a measured distance while being timed (distance/ time), can be evaluated in normal and fast pace	Criteria: 1–6	Validated, easy to perform, robust predictors of cognitive and motor decline and health outcomes including falls and mortality	Influenced by non CNS factors. May have ceiling effect in high functional people	Minimum significant change: 5cm/sec (121). Clinical significant change: 10 cm/sec	Included in core and minimum battery
**2. Dual-Task Gait**	Motor-divided attention task that requires individuals to walk while doing a cognitively demanding task	Criteria: 1–6	Isolates cognitive control from other determinants of gait, unmasks latent gait disturbances, possible to adapt difficulty levels of the gait and/ or cognitive task, ceiling effect	No consensus on which cognitive task to use; role of task prioritization needs to be determined	Not defined yet	Included in core and minimum battery
**3. Gait Variability**	The amount of stride-to-stride fluctuation in temporal and spatial parameters of gait	Criteria: 1–5		Requires instrumented methods	Minimum significant change: stance time and swing time *SD* = 0.01 s; step length *SD* = 0.25 cm (122)	Included in core battery; missing criteria 6
**4. Timed Up & Go (TUG**)	Seated on a chair individuals are asked to rise, walk 3m, turn around return to a seated position	Criteria: 1, 3–6	Provides info about rising, turning and transferring. Not sensitive to dual task interference	Floor effect, difficult to separate out components for biological studies.	>13.5 s high risk of falls (61). Clinical change ~2 s (1.5 *SD* of normative data).	Included in core battery; missing criteria 2
**5. Short Physical Performance Battery (SPPB**)	Assesses lower extremity functioning in older persons. Includes: repeated chair stands, balance tests, and a short walk	Criteria: 1, 3–6	Good composite measure. Correlates with cognitive test (MMSE, Digit Symbol Substitution (66), TMT B-A (67)) and with cognitive decline (68)	Ceiling effect	Clinical Significant change: 1.0 (123)	Included in core battery; missing criteria 2
**6. Berg Balance Scale (BBS**)	Evaluates functional balance performance	Criteria: 3–5	Correlates with TMT B (71). Sensitive to exercise intervention (124)	Ceiling effect, weak correlation with cognitive measures, and expertise required	Minimum clinical significance change depends on participant’s baseline score: 0–24 = 5 pts; 25–34 = 7 pts; 35–44 = 5 pts; 45–56 = 4 pts (125)	Not included; missing criteria 1,2, 6
**7. Five-Times- Sit-to-Stand (FTSS**)	Participants need to perform five complete sit-to-stand movements as fast as possible without using arms to rise from a chair	Criteria: 1, 3–6	Easy to perform. Sensitive to global cognitive impairment and mobility decline	Ceiling effect	>15–20 seconds to complete five movements may indicate global cognitive impairment	Not included; part of SPPB.

*Note*: CNS = Central nervous system; SD = Standard deviation.

#### Gait speed (usual and fast pace)

Gait speed is expressed as distance/time and is assessed by timing individuals while they walk a measured distance, which has been tested in the literature in the range of 2.4–8 m (18,19). Some studies have used longer distances (eg, 20 m), which are required to assess steady-state walking, but shorter distances are more practical in some contexts (eg, clinical). Slow gait speed predicts future fall risk, hospitalization, institutionalization (23,24), cognitive decline (25), incident dementia (12,26–28), and mortality (29). Gait speed is easy to measure, very sensitive to both motor and cognitive changes, and responsive to interventions. Gait speed measure protocols, normative values, and cut-offs are available but they are population dependant (24,29–31). In Supplementary Table B, proposed instructions and protocols are described. Gait speed presents ceilings effect only when used in high functioning individuals and it is influenced by non-central nervous system (CNS) factors, including musculoskeletal and cardiorespiratory diseases, among others. Additionally, gait speed tested as a fast pace can provide additional information, particularly in highly functioning people, and it has been proposed as a measure of physical and cognitive reserve (32,33).

#### Dual-task gait

Dual-task gait (DTG), defined here as walking while performing a cognitively demanding task, isolates the cognitive component of locomotion and provides insights into the mechanisms of motor control (1,34). Example of detailed instructions used by our CCNA group is presented in Supplementary Table B. Emerging evidence suggest that DTG serves as a robust marker of cognitive-motor interaction, is associated with cognitive performance (ie, low cognitive performance is associated with worsening DTG) and can predict cognitive decline, incident dementia, and falls (34–39). Currently, there is no consensus about which cognitive challenge task (eg, naming items/animals, calculations, reciting alphabet letters) should be used paired with walking or the predicted ability of one task over other. Thus, they should not be considered interchangeable. An excellent recent systematic review and meta-analysis found robust evidence that different cognitive tasks (reaction time, verbal fluency, mathematical, working memory, and metal tracking) in different populations (healthy and clinical) affect spatio-temporal gait parameters by decreasing speed, cadence, and stride length, an increasing stride time and stride time variability (17). Along with this meta-analysis, subsequent studies confirmed that dual-task-related changes in gait speed are sensitive and could distinguish groups of healthy participants from those with neurological disorders, even among those with mild deficits such as mild cognitive impairments and dementias (2,40,41). Accepted recommendations for choosing cognitive tasks include having a constant cognitive demand, quantifiable performance on the task that does not visually interfere with the gait path. Finally, instructions regarding prioritization of focus on the cognitive or gait task, or neither, should be provided based on the main research questions (42). Gait performance in dual-task testing can be expressed as a dual-task gait cost (DTGC = [(single-task gait – dual-task gait)/single-task gait gait] × 100) that adjusts for an individual’s baseline gait characteristics (12,34). A limitation of DTGC is that it may have ceiling effects, depending on the populations assessed (43). Dual-task cognitive cost (DTCC) provides a measure of the effect of the walking task over cognitive performance. Commonly, older adults prioritize gait (motor performance) over cognitive performance to maintain the “posture first” strategy (10). As such, DTCC may be larger than DTGC and would be missing information without the calculation of costs in both domains. However, dual-task gait is generally reported as changes in speed or DTGC, without accounting for DTCC cost, presumably for simplicity because the way to calculate the cognitive cost varies depending on the type of cognitive task used (ie, mental tracking or phonemic task) (17).

#### Gait variability

Gait variability quantifies fluctuations in temporal and spatial gait parameters. The gait parameters most studied have been stride-to-stride and step-to-step variability, both temporal and spatial, but variability in additional gait parameters has been also explored (44–47). Variability can be expressed simply as standard deviation, or as coefficient of variation which standardizes variability according to the mean. A low stride-to-stride variability reflects a rhythmic and stable gait, whereas high gait variability reflects an unstable walking pattern (46,47). Gait variability depends on gait speed showing a “U shape” pattern where very low (<0.4 m/s) or very high gait speed (>1.4 m/s) may increase variability (48–50), and thus are not recommended speeds to assess variability. High stride time variability is common in individuals with cognitive impairment (37), particularly those with low executive function (35,51), and it has been shown to be more sensitive to cognitive changes than other gait parameters (12). For instance, cognitively normal older adults have low stride time variability; however, high stride time variability has been described in Parkinson’s disease, Alzheimer’s disease, and has been associated with high risk of future falls, and mobility decline (28,52–54). It has been shown that abnormalities in gait variability are more evident during dual-task gait testing than in single-task (12).

A limitation is that gait variability measures require instrumented methods (electronic walkways, accelerometers, foot switches, or optoelectronic systems), and a minimum of approximately 12 steps is required to allow measurement of step-to-step variability during the preferred gait velocity (49). Still, it is interesting to note that even a standard Smartphone placed on the body can be used to quantify gait variability (55,56).

#### Timed up and go (TUG)

For this test, individuals are instructed to rise from a seated position, walk 3m at their usual pace, turn around and return to the seated position. The completion times on the TUG and TUG with a secondary cognitive task are related to measures of cognitive function, in particular executive function (57). It is validated and reliable, sensitive to cognitive and mobility impairments, and can provide information about rising, turning and transferring, everyday tasks important for the maintenance of mobility. TUG is not a good fall predictor and fall (58–60) prediction seems not to be enhanced by adding dual-task challenges, suggesting a low sensitivity to cognitive changes (61). Limitations of the TUG include the presence of a floor effect, and the difficulty in separating gait components from the overall measure since the TUG is a composite measure. Instrumented implementations of the TUG (iTUG) can address many of these limitations (62–64).

#### The Short Physical Performance Battery (SPPB)

The SPPB is a 15-minute objective assessment tool for evaluating lower extremity functioning in older people (65). It includes (1): repeated chair stands (2) balance tests, and (3) a 2.44 or 4 m walk. It is widely used and validated, simple to administer, and is considered a good composite measure of mobility (65). The SPPB test has been associated with performance in several cognitive measures including global cognition (Mini-Mental State Exam [MMSE] (66)), processing speed (DSST (66)), executive function and attention (66), and Trail-Making Test (TMT) B (67). Moreover, individual SPPB items (4m walking time and sit-to-stand time) predict the onset of cognitive decline in older people, particularly in women (68), and injurious falls (69). Its limitation is that is has ceiling effects.

#### Berg Balance Scale (BBS)

The BBS is a widely-accepted balance measure that was originally designed for stroke populations and used in rehabilitation settings (70). It consists of balance-related tasks, such as standing on one foot and standing up from a seated position. It has limitations, such as a well-defined propensity for ceiling effects, not providing sufficient gradient of dispersion in scores, and its performance is not clearly related to measures of cognitive function (57) except for a weak association with performance in TMT B after dual-task training intervention in individuals with history of multiple falls (71). This may suggest that the association between BBS and cognitive functioning may be very selective, indicating low sensitivity to cognitive changes (72).

#### Five-Times-Sit-to-Stand (FTSS)

This test includes five consecutive sit-to-stand movements done as fast as possible, without using arms as leverage. It is sensitive to global cognitive impairment (73) and mobility decline (74), although it has a ceiling effect. It takes approximately 3 minutes to perform.

### Cognitive Measures Appraised

Global cognitive tests and cognitive measures that underlie areas such as executive function, attention, episodic memory, and processing speed were considered (Table 3) based on previous systematic reviews showing consistent associations with mobility outcomes (4). Measures of additional cognitive domains, such as visuospatial abilities or verbal fluency, were not included in our candidate tests because they were less studied in the literature. However, there is evidence that both of these cognitive domains are associated with gait impairments (75) and increased risk of falls in prospective analyses (76).

**Table 3.** Cognitive Tests Appraised to Assess Motor-cognitive Interaction in Aging

Measure	Description	Prespecified Criteria Fulfilled	Advantages	Key Limitations	Clinical Significance of Change	Result
**1. Mini-Mental State Exam (MMSE**)	Assessment of general cognitive status of participants	Criteria: 1, 3, 4, 6	Widely used, not complex to administer	Ceiling effect, sensitive to interventions only if participants have below cut-off scores (124)	<27 pts risk of MCI; <25 pts risk of dementia (126)	Not included; missing criteria 2, 5
**2. Montreal Cognitive Assessment (MoCA**)	Assessment of general cognitive status of participants	Criteria: 1–6	Widely used, validated, not complex to administer. More sensitive than MMSE to detect MCI in the oldest old adults. Sensitive to predict dementia	None	≤25 pts risk of MCI; ≤24 risk of dementia (127)	Included in core and minimum battery
**3. Digit Symbol Substitution Test (DSST**)	Measures attention and executive function	Criteria: 1, 3–6	Very easy to perform and provides a good measure of processing speed and attentional capabilities. Sensitive to mobility decline (82)	Ceiling effect	Not defined yet	Included in core battery; missing criteria 2
**4. Trail Making Test part A and B (TMT A & B**)	Assessment of executive function	Criteria: 1, 3–6	Sensitive to attention, executive function deficits and motor decline (67,84)	Ceiling effect	Scores < or >1.5 *SD* relative to normative data indicate clinical change	Included in core and minimum battery
**5. Stroop Test**	Measures executive function by assessing the ability to inhibit an automatic response	Criteria: 1–5	Correlated with gait variability enhanced by dual task performance (86,87), very responsive to interventions like aerobic exercise (88,128) and resistance training (90,91)	Full versions are time consuming to administer	Not defined yet	Included in core battery; missing criteria 6
**6. Rey Auditory Verbal Learning Test (RAVLT**)	Assessment of episodic memory (129–132)	Criteria: 1, 3–6	Sensitive to short-term auditory-verbal memory, rate of learning, retention of information, and differences between learning and retrieval	Time consuming to administer	Not defined yet	Included in core battery; missing criteria 6
**7. Mood measures**	Questionnaires assessing mental health, specifically anxiety and depression. The Patient Health Questionnaire-9 (PHQ9) (97), the Geriatric Depression Scale (GDS-30) (98), the Cornell Scale for Depression in Dementia (CSDD) (99), and the Generalized Anxiety Disorder Scale (100)	Criteria: 1, 4, 5	Depression and anxiety are correlated with mobility measures such as gait speed and variability	Floor and ceiling effects, requires recollection of facts, not validated in individuals with memory and mobility impairments	No minimum clinical significant change, each test scale indicates degree of symptom severity	Not included; missing criteria 2, 3, 6

*Note*: CNS = Central nervous system; MCI = Mild cognitive impairment; SD = Standard deviation.

#### The Mini-Mental State Exam (MMSE)

This test is an assessment of general cognitive status that includes components of temporal and spatial orientation, memory recall, and ability to follow simple instructions. It is widely used, validated for motor-cognitive interaction (66), and easy to administer, taking between 5 and 10 minutes to complete. However, it has a clear ceiling effect and its sensitivity to monitor interventions is low.

#### Montreal Cognitive Assessment (MoCA)

MoCA is considered a reliable assessment of global cognition, with a good sensitivity and specificity for screening mild cognitive impairment (MCI) and dementia (77). It assesses visuospatial skills, memory recall, executive function, attention, language and verbal fluency, and temporal and spatial orientation. Emergent research links MoCA performance with mobility. Individuals with higher MoCA scores have lower fall risk profiles (78) and better TUG times (79). Low performance in the MoCA test (MoCA<26) is associated with poor gait performance in single and dual-tasking (12,41) and increased risk of falls over the next year (80). A potential limitation, as in other global cognitive measures, is that MoCA might have limited capability to detect changes across time and longitudinal data are less available (81). MoCA test has been coupled with gait speed, described as gait and cognition syndrome, and shown to increase the risk of progression to dementia by seven times in community older adults (6).

#### Digit Symbol Substitution Test (DSST)

In this test, the participant is a given a list of digits and symbols in which each symbol corresponds to a digit. Then, the participant is asked to write down the appropriate symbol on a list of digits, as fast as possible. The DSST is part of the WAIS Wechsler tests, easy to perform, and an accepted measure of processing speed and attentional capabilities. Previous studies have consistently shown that low DSST performance is associated with faster decline in gait speed (82) and worse performance in dual-task tests (66). It has been also associated with mobility decline and future disability. However, it has a ceiling effect in high functioning populations (83).

#### Trail-Making Test (TMT) A & B

In the Trail Making Test A (TMT A), participants are instructed to draw a line connecting numbers in ascending order, from 1 to 25. In the Trail Making Test B (TMT B), participants have to alternate between numbers and letters, connecting them both in ascending order. TMT A assesses visual attention and cognitive speed skills whereas TMT B evaluates higher-order cognitive skills such as working memory and task-shifting (mental flexibility). TMT B-A provides the processing time required to switch between task rules (number to letters or vice-versa) and is thought to represent attentional-switching. TMT B-A is very well correlated with gait performance, especially in complex environments with obstacle avoidance (43,84,85).

#### The Stroop Test

The Stroop Test is designed to measure the ability to inhibit an automatic response. Participants are asked to provide speedy responses to the color of the ink in the stimuli presented. Reaction times to three Stroop levels are recorded: (a) color only, (b) color words that are printed in ink that matches the word (ie, BLUE in blue ink), and (c) color words that do not match the ink color (interference condition, RED printed in blue ink). This task is well correlated with gait performance, particularly gait variability during dual-task gait (86,87). The Stroop test is responsive to interventions such as aerobic exercise (88,89) and resistance training (90,91). A limitation of the Stroop test full version is that is time consuming to administer.

#### Rey Auditory Verbal Learning Test (RAVLT)

This test measures episodic verbal memory, where subjects are given a list of 15 words that they have to repeat over 5 consecutive trials. Subsequently, they are asked to remember an interference list of words, and lastly they are asked to remember the words of the first list in a short recall (after 5 minutes) and in a delay recall trial (after 20 minutes). RAVLT is one of the few memory test that has been associated with gait performance and used as outcome to show that slow gait predict decline in RAVLT scores (92). RAVLT is affected by sex and education and that it is time consuming to administer (93).

#### Mood measures

Although mood measures were not found to be consistently associated with mobility measures in our scoping review, measures of depression and anxiety were considered because two position background articles suggested that mood should be evaluated when assessing motor-mobility decline in aging (2,9). Depressive symptoms are known to influence cognitive outcomes (94), gait performance (95), and fall risk (96). Tests considered were Patient Health Questionnaire-9 (PHQ9) (97) as a self-rated diagnostic measure for depression, the Geriatric Depression Scale (GDS-30) (98), the Cornell Scale for Depression in Dementia (CSDD) (99), and the General Anxiety Disorder scale (100). These tests have floor and ceiling effects, and there is a lack of evidence showing sensitivity to mobility performance or cognitive-motor interaction.

### Rationale for the Selected Tests and Their Role for Diagnosis, Prognosis, or as Outcomes

Tests selected for the core-battery had to fulfil at least five out of the six prespecified criteria (Table 4; Figure 2). Tests from the core-battery that fulfilled all six criteria were also included in the minimum-battery (Table 4). Tests that presented two or more limitations were excluded from both batteries.

Based on the literature, we used a Likert scale to compare the selected tests by their use in clinical or research settings, their application as diagnostic, prognostic or outcome tools, as well as their association with the cognitive-motor interaction. We also reported the highest effect size found in the literature for the association between these mobility and cognitive tests (Table 5).

**Table 5.** Comparison of the Selected Tests by Their Use in Clinical/Research Setting, and Their Application as Diagnostic, Prognostic, and Outcome Purposes with Their Highest Reported Effect Size

	Clinical Setting	Research Setting	Diagnostic	Prognostic	Outcome	Cognitive-Motor Interaction	Highest Effect Size Reported
**Cognition**							^a^
MoCA^c^	+++	+	+++	++	+	++	**Gait speed**: 0.43 (6) **Gait variability**: 0.04 (27) **DTG**: 0.47 (27) **SPPB**: 0.74 (27)
RAVLT	+	++	+++	++	+	+	**Gait speed:** 0.29 (6) **Gait variability**: 0.12 (27) **DTG**: 0.71 (27) **SPPB**: 0.25 (27)
TMT A & B	++	+++	++	+++	+++	++	**Gait speed**: 0.39 (133) **Gait variability**: 0.05 (35) **DTG**: 0.68 (134) **SPPB**: 0.47 (27)
DSST	+	+++	+	+++	++	++	**Gait speed**: 0.51 (66) **Gait variability**: 0.46 (113) **DTG**: 0.45 (133) **SPPB**: 0.44 (113)
Stroop Test	+	+++	++	+	++	+	**Gait speed**: 0.59 (135) **Gait variability**: 0.05 (35) **DTG**: 0.68 (134)
**Mobility**							^b^
Gait speed^c^	++	+++	+	+++	++	++	**Global cognition**: 0.41 (135) **Executive function**: 0.59 (135) **Memory**: 0.22 (136) **Processing speed**: 0.45 (66)
Gait variability	-	+++	+	+	+	++	**Global cognition**: 0.04 (27) **Executive function**: 0.05 (35) **Memory**: 0.12 (27) **Processing speed**:0.62 (27)
Dual-task Gait	+	+++	+++	++	+	+++	**Global cognition**: 0.47 (27) **Executive function**: 0.56 (66) **Memory**: 0.71 (27) **Processing speed**: 0.45 (66)
TUG	+++	++	++	++	+	+	**Global cognition**: 0.46 (135) **Executive function**: 1.04 (135)
SPPB	+	+++	+	+	+	+	**Global cognition**: 0.41 (137) **Executive function**: 0.47 (27) **Memory**: 0.25 (27) **Processing speed**: 0.25 (27)

*Note*: The magnitude of the associations for each category is presented using a Likert scale from + to +++, based on the scoping review.

DSST = Digit Symbol Substitution Test; MoCA = Montreal Cognitive Assessment; RAVLT = Rey Auditory Verbal Learning Test; SPPB = Short Physical Performance Battery; TMT = Trail Making Test; TUG = Timed-up and go.

^a^Effect sizes for associations between each cognitive test and gait speed, gait variability, dual-task gait (DTG), TUG, and repeated chair stands (from SPPB). ^b^Effect sizes for associations between each mobility test and global cognition, executive function, memory, and processing speed (19). ^c^Gait speed at usual pace is used in the Motoric Cognitive Risk Syndrome coupled with subjective cognitive complains, and in the “Gait and Cognition Syndrome” coupled with the MoCA test.

### Core-battery of Tests Selected

The following tests were selected for the core-battery: gait speed (normal and fast-paced), dual-task gait, gait variability, TUG (single-task), and SPPB (Table 4) since they fulfilled at least five out of the six pre-established criteria. These tests have been previously validated for evaluating changes in mobility and cognitive performance, are applicable in clinics and across research studies, and are sensitive to interventions. Regarding cognitive assessments, experts determined that the most suitable measures to be included in the core-battery of cognitive tests were MoCA test, digit symbol substitution, TMT A and B, Stroop test, and RAVLT (Table 4), based on the fulfillment of at least five of the pre-established criteria. These cognitive tests are feasible, inexpensive, easy to perform, and commonly implemented in research and clinical practice.

### Minimum-battery of Tests Selected

For the minimum-battery, gait speed and dual-task gait were retained from the mobility tests because they fulfill all prespecified criteria (Table 4). Both tests consistently show strong association with cognitive performance and decline, and they can be performed in less than 5 minutes without special expertise or training (12,26–28).

**Table 4.** Proposed “Core-battery” and “Minimum-battery” of Tests

Core-battery of Tests	
Mobility tests	Cognitive tests
Gait speed (normal and fast pace)	MoCA
Dual-task gait (speed)	TMT A and B
Gait variability	Digit Symbol Substitution
Timed Up and Go	Stroop test
SPPB	RAVLT
**Minimum-battery of Tests**	
Mobility tests	Cognitive tests
Gait speed (normal pace)	MoCA
Dual-task gait (speed)	TMT A and B

*Note*: MoCA = Montreal Cognitive Assessment; RAVLT = Rey Auditory Verbal Learning Test; SPPB = Short Physical Performance Battery; TMT = Trail Making Test.

Regarding cognitive tests, MoCA and TMT (A & B) were retained (Table 4) because they met all prespecified criteria, they have extensively shown to be sensitive to cognitive and mobility performance and to predict mobility decline, and importantly, in the case of TMT, to also predict falls. MoCA has the advantage that it can be reported not only as a total score but also considered in terms of as subscores for independent cognitive domains (101). Both, MoCA and TMT, are easy to perform in less than 10 minutes. In addition, the reporting of cognitive complains provides helps in characterization of MCR syndrome, and due to its simplicity it is suggested to be recorded in conjunction with the rest of the core and minimum battery of scores.

The four tests retained for the minimum-battery have shown to be sensitive to both pharmacological (eg, cognitive enhancers, amphetamines) and nonpharmacological (eg, physical exercises and cognitive training) interventions targeting cognition that motor performance and gait (102–106). Specifically, three of the four selected tests have also been included in other guidelines for cognitive assessments, including those developed for vascular cognitive impairment by the NIH and Canadian Stroke Network Consensus (107) (MoCA test) and the NIH tool box (TMT and gait speed).

## Discussion

A new paradigm is emerging in which mobility and cognitive impairments are treated as interrelated entities in aging and neurodegeneration, requiring an integrated approach to measure both domains. This consensus aimed to identify a complementary battery of existing tests of mobility and cognition in community-dwelling older adults, which may accelerate the study of dementia, falls, and related disabilities in community-dwelling older adults, in research and clinical settings.

The core and minimum-battery of tests that we identified have been mainly used for risk prediction of cognitive and/or mobility decline. Some attempts have been made to use them for diagnosis purposes as quantitative gait measures that can define cognitive profiles (12,108), as shown in Table 5. Additionally, when these cognitive and motor tests were combined, as seen in MCR or Gait and Cognition syndrome constructs, they better predicted cognitive or mobility decline and incident dementia (6,12,109). Mounting evidence supports that some of them, such as gait speed, are sensitive outcomes to interventions deemed to improve cognition and mobility (104,110–113). This selected battery of tests are adding to previous steps taken to integrate and focus the study of motor-cognitive interactions (2,114,115) and are aligned with previous systematic reviews appraising tests for mobility and cognition interaction (18,19). Facilitating the dissemination and harmonization of appropriate tests will boost comparisons between studies and interventions, and ultimately may enhance the advancement in the field and our understanding of the motor-cognitive interaction. The core-battery may be more appropriate for specific research regarding motor-cognitive outcomes because it includes a more detailed and sometimes technological-dependent testing to investigating motor-cognitive interactions. In addition, the core-battery tests are all free and have unrestricted access. On the other hand, the minimum-battery can easily be implemented in clinical practice and in large epidemiological studies in less than 15 minutes with no sophisticated equipment or specialized training.

Implementation of these batteries will also help to answer additional research questions, for example, what are the best mobility measures that accurately predict the development of different subtypes of cognitive impairments and dementia (Alzheimer’s dementia and non-Alzheimer’s) in community older adults. For example, it has been shown that gait changes and dual-task gait changes can differentiate between subjects with MCI and mild AD; worse performance in dual-task gait during demanding cognitive challenges, like serial subtraction by 7s, has been associated with specific impairments in attention, executive function, working memory, and episodic memory in MCI (41,116). It has been also shown that gait can vary between amnestic and non-amnestic type in MCI (12,117). Recently, quantitative gait performance in subjects fulfilling criteria for MCR helped detection of subtypes of cognitive impairment (26,109).

It is possible that technological advancements may soon allow more sophisticated analysis of mobility or cognition, especially in real-life environments, for application to clinical or epidemiological investigations, as the use of wearable sensors are becoming more available (118). Accelerometer-based wearable sensors can be utilized to quantify common clinical parameters of gait, including gait speed and step-to-step variability (119). Although relatively new in the clinical setting, these inexpensive and highly portable technologies have been proven reliable and valid in a controlled setting, and are showing great promise for separating the effects of mobility and cognitive impairments on gait function (120). As newer measures become more standardized and accessible, they may be considered for incorporation into the harmonized battery of measures in the future.

The current batteries recommended are not without limitations and should be interpreted in the context that a consensus process is not a completely objective exercise. The original selection of the candidate measures was done based on a scoping review by one member and a comprehensive systematic review was not performed. The panel of experts, including our advisory board, while members of our *Canadian Gait and Cognition Network*, may not share the opinions of all potential users of the mobility and cognitive measures selected. While attempting to account for practice-related issues, the panel’s expertise was skewed towards research-related issues. There may still be questions about applicability in some populations and/or settings; however, we focused on community-dwelling older adults free of overt neurological diseases. Future directions may include to better address which tests are better suited for diagnosis, prognosis or outcome measures, ethnic differences on the selected measures, and specifying the role of sex and gender.

## Funding

The CCNA MEC team is funded by Canadian Consortium on Neurodegeneration in Aging (CCNA – Grant# “FRN” CNA 137794) which receives funding from the Canadian Institutes of Health Research and other partner organizations.

## Conflict of Interest

M.M.O., J.H., C.R., S.S., and J.V. serve on the editorial board of the Journal of Gerontology: Medical Sciences. All other authors declare they have no conflicts of interest.

## Supplementary Material

gly148_suppl_Supplementary_Table_A

gly148_suppl_Supplementary_Table_B
