# Supplementary material for: Consensus on Shared Measures of Mobility and Cognition: From the Canadian Consortium on Neurodegeneration in Aging (CCNA)
Source: J Gerontol A Biol Sci Med Sci. 2018 Jun 21;74(6):897–909. doi: 10.1093/gerona/gly148 (PMC6521916; doi:10.1093/gerona/gly148)
Supplement: gly148_suppl_Supplementary_Table_A [file gly148_suppl_supplementary_table_a.docx]

# Supplementary Table A. Consensus members and international advisory board.

|  | Consensus Members and Speakers | | | | | |
| --- | --- | --- | --- | --- | --- | --- |
| Participant | | City/Province | Country | Primary Appointment | Subject area expertise | Contributions to the Consensus |
| M. Montero-Odasso | | London,  Ontario | Canada | Professor of Medicine | Geriatric medicine; gait and cognition | Consensus Chair. Drafted white paper with scoping review. Oversaw logistic of round of consultation and drafted first program of consensus meeting. Chaired the rounds of consultation for the white paper and the round table debate |
| R. Camicioli | | Edmonton,  Alberta | Canada | Professor of Neurology | Geriatric neurology; gait and cognition | PRESENTATION TOPIC: Mobility measures on aging pertinent to mobility and cognition. General mobility measures |
| Q. Almeida | | Waterloo,  Ontario | Canada | Professor of Kinesiology | Parkinson disease; gait and cognition | PRESENTATION TOPIC: Quantitative temporal and spatial measures of gait in older people and neurodegenerative diseases |
| K. Li | | Montreal,  Quebec | Canada | Professor of Psychology | Cognition; cognitive training | PRESENTATION TOPIC: Cognitive considerations in the assessment and improvement of dual-task mobility and cognition in older people |
| S. Fraser | | Ottawa,  Ontario | Canada | Professor of Health Sciences | Gait and cognition | PRESENTATION TOPIC: Impact of exercise on cognition and psychological well-being in older adults populations |
| L. Bherer | | Montreal,  Quebec | Canada | Professor of Psychology | Exercises, mobility and cognition | PRESENTATION TOPIC: Impact of exercise on cognition and psychological well-being in older adults populations |
| T. Liu-Ambrose | | Vancouver, British Columbia | Canada | Professor of Physical Therapy | Exercises, mobility and cognition | PRESENTATION TOPIC: Targeted exercise training, cognition and mobility. |
|  | Consensus Members | | | | | |
| Participant | | Province | Country | Primary Appointment | Subject area expertise | Contributions to the Consensus |
| A.M. Burhan | | London,  Ontario, | Canada | Chair, Division of Geriatric Psychiatry | Geriatric psychiatry; mood disorders | Part of the rounds of consultation for the white paper and part of the round table debate |
| J. Doyon | | Montreal,  Quebec | Canada | Professor of Psychology | Imaging; motor learning | Part of the rounds of consultation for the white paper and part of the round table debate |
| S. Muir-Hunter | | London,  Ontario, | Canada | Assistant Professor of Physical Therapy | Gait and falls | Part of the rounds of consultation for the white paper and part of the round table debate |
| W. McIlroy | | Waterloo,  Ontario | Canada | Professor of Kinesiology | Quantitative gait and balance | Part of the rounds of consultation for the white paper and part of the round table debate |
| L. Middleton | | Waterloo,  Ontario | Canada | Assistant Professor of Kinesiology | Kinesiology | Part of the rounds of consultation for the white paper and part of the round table debate |
| J. Morais | | Montreal,  Quebec | Canada | Associate Professor of Medicine | Geriatric medicine | Part of the rounds of consultation for the white paper and part of the round table debate |
| R. Sakurai | | London,  Ontario | Canada | Fellow of Department of Medicine | Motor learning; fear of falling | Part of the rounds of consultation for the white paper and part of the round table debate |
| M. Speechley | | London,  Ontario | Canada | Professor of Epidemiology and Biostatistics | Epidemiology of aging; falls | Part of the rounds of consultation for the white paper and part of the round table debate |
| A. Vasudev | | London,  Ontario | Canada | Assistant Professor of Geriatric Psychiatry and Medicine | Geriatric psychiatry; mood disorders | Part of the rounds of consultation for the white paper and part of the round table debate |
|  | International Advisory Board | | | | | |
| Participant | | City/Province | Country | Primary Appointment | Subject Expertise | Contributions to the Consensus |
| J. Verghese | | Bronx,  New York | USA | Professor of Neurology | Geriatric neurology; gait and cognition | Part of the rounds of consultation for the white paper |
| S. Studenski | | Pittsburgh, Pennsylvania | USA | Senior Investigator | Geriatric medicine and neurology; CNS; mobility | PRESENTATION TOPIC: Dysmobility and the Relationship with Cognition. The value of gait speed |
| O. Beauchet | | Angers,  Angers | France | Professor of Medicine & Neurology | Geriatric medicine; gait and cognition | PRESENTATION TOPIC: Gait and dual-tasking. A paradigm to explore the mobility and cognition in older people? |
| J. Hausdorff | | Tel Aviv, Tel Aviv / Boston, Massachusetts | Israel, USA | Professor of Medicine | Geriatric neurology; mobility and cognition | Part of the rounds of consultation for the white paper |
| C. Rosano | | Pittsburgh, Pennsylvania | USA | Professor in Epidemiology | Neuroscience; brain imaging | PRESENTATION TOPIC: Imaging on mobility and cognition |

CNS: Central Nervous System
