# Supplementary material for: Consensus on Shared Measures of Mobility and Cognition: From the Canadian Consortium on Neurodegeneration in Aging (CCNA)
Source: J Gerontol A Biol Sci Med Sci. 2018 Jun 21;74(6):897–909. doi: 10.1093/gerona/gly148 (PMC6521916; doi:10.1093/gerona/gly148)
Supplement: gly148_suppl_Supplementary_Table_B [file gly148_suppl_supplementary_table_b.docx]

Supplementary Table B. Detailed instructions used in the gait assessment of the CCNA cohort.

| **Walking Task** | **Instructions** |
| --- | --- |
| Preferred or usual gait | "When I say GO, please walk at your usual pace in a comfortable and safe way until you cross this line [INDICATE END LINE]." |
| Counting backwards | “When I say GO, please walk at your usual pace and at the same time count backwards from 100 by 1s, out loud, until you cross this line [INDICATE END LINE]. Remember that it is important that you do not stop your walking or counting.” If participant have difficulties understanding, evaluators are allowed to clarify by providing a verbal example: "For example 100, 99, 98, ... and so on". Evaluators are allowed to prompt the tasks if participants tend to stop during the walk. |
| Naming animals | “When I say GO, please walk at your usual pace and at the same time try to name as many different animals as you can think of out loud, until you cross this line [INDICATE END LINE]. Remember that it is important that you do not stop your walking or talking.” |
| Serial sevens | “When I say GO, please walk at your usual pace and at the same time count backwards from 100 by 7s, out loud, until you cross this line [INDICATE END LINE]. Remember that it is important that you do not stop your walking or counting.” Evaluators are allowed to prompt the tasks if participants tend to stop during the walk. |
| Fast gait | “When I say GO, please walk as fast as you can, as safe as you can, and without running, until you cross this line [INDICATE END LINE].” |
